# Balance Control is Sequentially Correlated with Proprioception, Joint Range of Motion, Strength, Pain, and Plantar Tactile Sensation Among Older Adults with Knee Osteoarthritis

**DOI:** 10.1186/s40798-024-00735-3

**Published:** 2024-06-09

**Authors:** Peixin Shen, Simin Li, Li Li, Daniel T. P. Fong, Dewei Mao, Qipeng Song

**Affiliations:** 1 College of Sports and Health, Shandong Sport University, Jinan, 250102 China; 2 Wolfson School of Mechanical, Electrical and Manufacturing Engineering, Loughborough University, Leicestershire, LE11 3TU UK; 3 Department of Health Sciences and Kinesiology, Georgia Southern University, Statesboro, 30460 USA; 4 School of Sport, Exercise and Health Sciences, Loughborough University, Leicestershire, LE11 3TU UK

**Keywords:** Berg Balance Scale, Fall, Risk Factors, Multivariate Linear Regression

## Abstract

**Background:**

Patients with knee osteoarthritis (KOA) are at high risk for falls, which is attributed to their impaired balance control. Identifying factors associated with balance control facilitates the development of precise KOA rehabilitation programs. This study was to investigate the correlations of balance control with proprioception, plantar tactile sensation (PTS), pain, joint range of motion (ROM), and strength among older adults with and without KOA, as well as the magnitudes and sequence of correlation of these factors to balance control.

**Methods:**

A total of 240 older adults with (*n* = 124, female: 84, age: 68.8 ± 4.0 years) and without (*n* = 116, female: 64, age: 67.9 ± 3.5 years) KOA were recruited and assigned to the KOA and control groups. Their proprioception, PTS, pain, ROM, and strength were measured. Pearson or Spearman correlations were used to test whether they were significantly related to their Berg Balance Scale (BBS), and factor analysis and multivariate linear regression were used to determine the degrees of correlation between each factor and the BBS.

**Results:**

Compared to the control group, the KOA group had lower BBS score, larger proprioception and PTS thresholds, smaller ROM, and less strength (p: 0.008, < 0.001–0.016, < 0.001–0.005, < 0.001–0.014, and < 0.001–0.002, respectively). In the KOA group, the BBS was weakly to moderately correlated with proprioception, PTS, pain, ROM, and strength (r: 0.332–0.501, 0.197–0.291, 0.340, 0.212–0.508, and 0.236–0.336, respectively). While in the control group, the BBS was correlated with proprioception and strength (r: 0.207–0.379, and 0.212–0.410). In the KOA group, BBS = 54.41+ (0.668*strength) - (0.579*PTS) - (1.141*proprioception) + (1.054* ROM) - (0.339*pain). While in the control group, BBS = 53.85+ (0.441*strength) - (0.677*proprioception).

**Conclusion:**

Worse proprioception and PTS, smaller ROM, and less strength were detected among older adults with KOA, and their proprioception, PTS, pain, ROM, and strength were all related to balance control. Proprioception had the strongest correlations, followed by ROM, strength, pain, and PTS. Precise KOA rehabilitation programs may be proposed following the sequence of improving the five factors.

## Key points

• Worse proprioception and plantar tactile sensation, smaller joint range of motion, and less strength were detected among older adults with knee osteoarthritis.

• Among older adults with knee osteoarthritis, balance control was sequentially correlated with proprioception, joint range of motion, strength, pain, and plantar tactile sensation.

• Precise knee osteoarthritis rehabilitation programs may be proposed following the sequence of improving proprioception, joint range of motion, strength, pain, and plantar tactile sensation.

## Introduction

Falls among older adults result in moderate to severe injuries, loss of independence, and even death [1]. The incidence of falls is 33% among older adults aged over 65 [2] and increases to 50% among those of the same age with knee osteoarthritis (KOA) [3]. Balance control is critical for maintaining postural stability and avoiding falls during functional activities [4], while the impairment of balance control is one of the strongest risk factors for falls [5]. Individuals with KOA have impaired balance control, which may be the main reason for their increased risk of falls [6]. The Berg balance scale (BBS) is clinically used to assess balance control among patients with KOA [7].

Worse proprioception [8] and plantar tactile sensation (PTS) [9], pain [10], less strength [11], and limited joint range of motion (ROM) [12] were detected among patients with KOA compared to their age-matched counterparts. The five factors are all potential determinants of balance control impairment. Proprioception refers to the perception of one’s body segments through information generated inside the body, which directly influences the control of locomotion [13]; PTS sensed by receptors in the foot sole skin can sense stimuli from outside the body, such as physical features of terrain [14]; Pain may affect muscle activation and increase postural sway, resulting in worse balance control [15]; Strength represents the ability of muscles to generate adequate corrective torques when the body is perturbed [16]; Joints cannot be fully extended or flexed to maintain balance when disturbances occur due to limited ROM [17].

Although it has been known that all five factors may be related to balance control, few studies have explored the magnitude and sequence of their correlation with balance control. Identifying which of the five factors is more relevant to balance control helps us prioritize treatment goals and develop effective rehabilitation programs. Moreover, multiple types of exercise therapy have been developed to improve balance control and reduce fall risks among patients with KOA [18], and their effects were attributed to the improvement of strength [19], proprioception [20], ROM, or pain [21]. However, it is unclear which of the improvements is more beneficial. Clarifying these issues can facilitate the development of accurate KOA rehabilitation programs and lead to a deeper understanding of the mechanisms by which falls occur among patients with KOA.

**Fig. 1:**
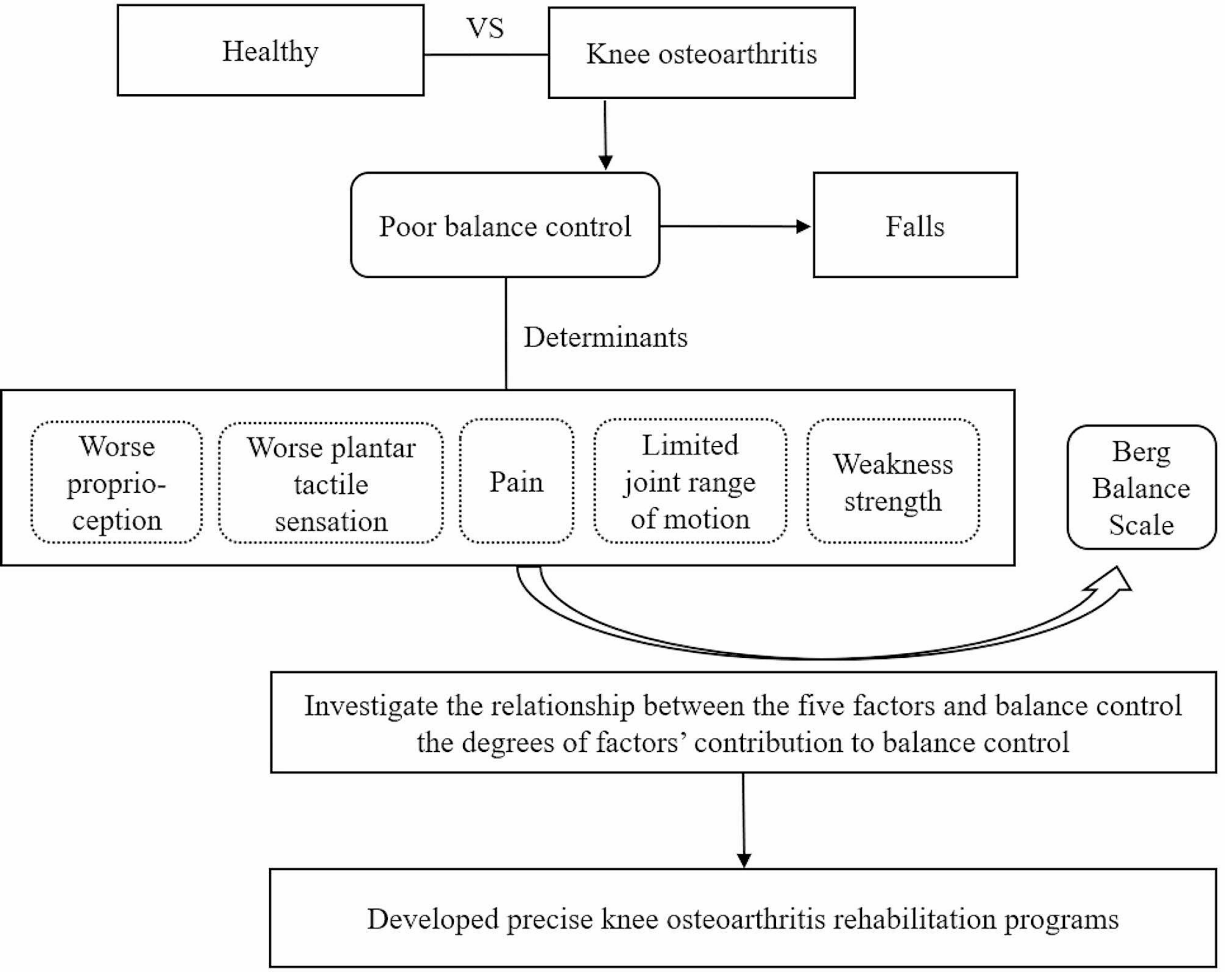
A roadmap of the study

This study aimed to investigate the relationship between the five factors and balance control and to explore the magnitude and sequence of their correlation with balance control (Fig. 1). It is hypothesized that: (1) Compared with older adults without KOA, KOA patients have poorer proprioception and PTS, smaller ROM, and less strength; (2) BBS is significantly correlated with these five factors; (3) The five factors contribute equivalent to BBS.

## Materials and Methods

### Study Design

This cross-sectional study conducted a comprehensive correlation analysis of multiple factors to balance control among individuals with and without KOA. The data collection was conducted from March 2021 to November 2022. The project was approved by the Ethics Committee of Shandong Sport University (No. 2021018) and was in accordance with the Declaration of Helsinki. All the participants signed informed consent before participating.

### Sample Size Estimate

Previous studies showed that the r^2^ of proprioception, strength, and tactile sensation to BBS were 0.175, 0.117, and 0.053 among older adults [22]. An *a priori* power analysis (G*Power Version 3.1) indicated that a sample size of 113 in each group is sufficient for regression with 5 independent variables to obtain the alpha level of 0.05 and the statistical power of 0.80 based on the minimum above-mentioned r^2^ (0.053 of tactile sensation).

### Participants

**Fig. 2:**
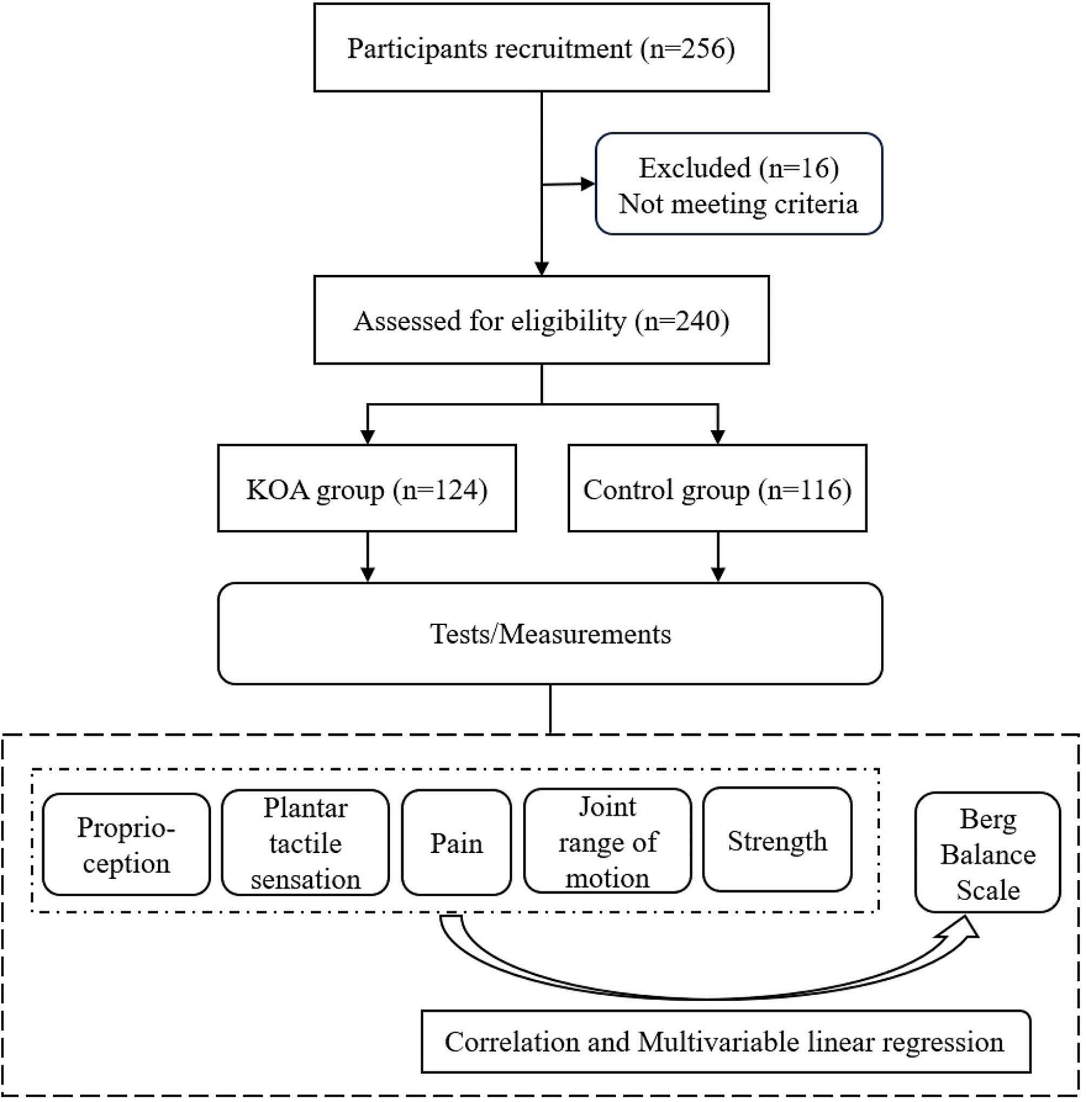
Participation flow chart. Final analysis included data from 240 participants

Participants were recruited in local communities in Jinan, China, through the distribution of flyers and delivery of presentations. A total of 256 people showed willingness to participate in the study. After the assessment, 240 met the inclusion criteria, of whom 124 had KOA (female = 84 and male = 40; right knee affected = 65 and left knee affected = 59; age: 68.8 ± 4.0 years; height: 160.6 ± 7.7 cm; body mass: 69.3 ± 10.1 kg; body mass index (BMI): 26.8 ± 3.2 kg/m^2^) and 116 did not have KOA (female = 72 and male = 44; age: 67.9 ± 3.5 years; height: 162.4 ± 7.7 cm; body mass: 64.5 ± 9.8 kg; BMI: 24.4 ± 3.3 kg/m^2^). The inclusion criteria were: (1) 65 years or older; (2) older adults with KOA were diagnosed by the same orthopedic surgeon as having unilateral, mild to severe KOA (graded of 1–4 by the Kellgren/Lawrence scale) based on X-ray; (3) older adults without KOA were diagnosed with no KOA or any other type of arthritis. Exclusion criteria were: (1) bilateral KOA; (2) lower extremity neurological or neuromuscular disease; (3) history of lower extremity joint surgery or fracture within three months before the study; (4) use of assistive walking devices; and (5) severe cognitive impairment. The participation flowchart is shown in Fig. 2.

### Protocols

All the participants completed a battery of tests, their proprioception, PTS, pain, ROM, and BBS were measured in a random order, and the strength was tested lastly to avoid fatigue.

### Proprioception Test

**Fig. 3:**
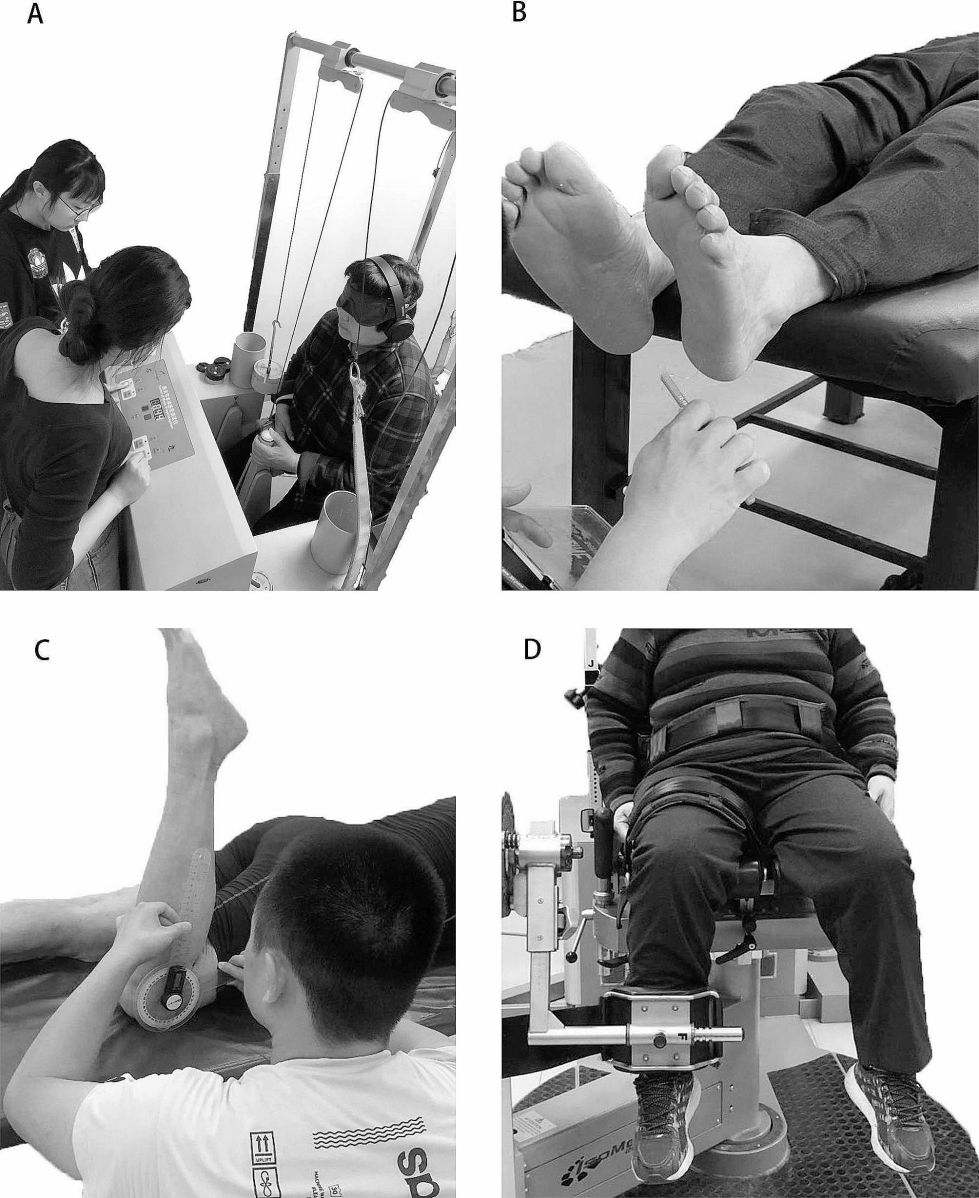
Test illustrations. **(A)** proprioception test using proprioception test devices, **(B)** PTS test with a set of Semmes-Weinstein monofilaments, **(C)** joint range of motion test using a universal goniometer, and **(D)** strength test using a IsoMed 2000 strength testing system

The proprioception thresholds (kinesthesia) on each participant’s affected knee and ankle were measured by a set of proprioception test devices (Sunny, AP-II, China), which showed good test-retest reliability at knee (ICC: 0.923–0.946) [23] and ankle [24] (ICC: 0.737–0.935). Dorsiflexion/plantarflexion at the ankle and flexion/extension at the knee were randomly tested. Each device comprises an operating platform and two pedals. Participants sat in a height-adjustable chair with both feet positioned on the testing pedal, hips and knees flexed at 90°, and ankles in a neutral position. Participants wore eye masks and noise-canceling headphones throughout the tests to minimize distractions. During each test, one of the pedals rotates at an angular velocity of 0.4°/s. As soon as the passive motion was perceived, participants immediately pressed a hand-held switch to stop the pedal (Fig. 3a). The proprioception threshold was defined as the angle of pedal rotation when the passive motion was perceived. Three trials were taken in each direction.

### PTS Test

A set of Semmes-Weinstein monofilaments (six-piece foot kit, North Coast Medical, Inc., Morgan Hill, CA, United States) with high test-retest reliability (ICC: 0.83 to 0.86) [25] were used to test the participants’ PTS of the affected leg while they were lying supine on a treatment table (Fig. 3b). The six sizes of monofilament used in this study were 2.83, 3.61, 4.31, 4.56, 5.07, and 6.65, with forces of 0.07, 0.4, 2, 4, 10, and 300 g applied when pressed into a C-shape (bent 90°). The filaments were randomly applied to the skin on the bases of the great toe, 1st and 5th metatarsal heads, arch, and heel, for 1 s with two repetitions. Randomized null stimuli were added to ensure that the participants could not anticipate the application of the filaments [26]. The test started with the thinnest filament and gradually with the thicker ones until the participant was able to detect the touch [26]. The participants were asked to respond verbally to the localization of the area under test when they perceived the stimulation. The sensitivity threshold was determined by the minimum monofilament gauge detected correctly. A larger gauge indicates a worse PTS.

### Pain Assessment

The pain score was measured by the Visual Analogue Scale (VAS), which has excellent reliability (ICC = 0.97) [27]. Zero points represented “no pain”, whereas 10 points represented “the worst pain possible”. A higher score indicates more severe pain.

### Range of Motion (ROM) Measurement

The ROM of the affected lower extremities was measured using a universal goniometer (Zimmer Ltd, Blackpool, UK), which showed excellent reliability (ICC of the hip [28], knee [29], and ankle [30]joints were greater than 0.90) (Fig. 3c). During the hip, knee, and ankle ROM measurements, the center of the goniometer was placed at the greater trochanter, fibular tuberosity, and heel, the fixed arm was placed along the long axis of the trunk, femur, and fibula, and the moving arm was placed along the long axis of the femur, fibula, and fifth metatarsal, respectively. Active ROM is measured, participants were instructed to flex or extend their hips, knees, and ankles as far as they could and hold them for 1–2 s without the assistant of anyone else. Three trials were conducted, and a higher score indicates a larger ROM.

### Strength Measurement

Strength of the hip, knee, and ankle flexors and extensors of the affected leg was measured using a strength testing system (IsoMed 2000, D. & R. Ferstl GmbH, Hemau, Germany) (Fig. 3d), which showed good test-retest reliability (ICC = 0.77–0.98) [31]. A five-minute warm-up was performed before the test began. During the knee test, participants were seated with their more affected knees and hips placed at 90° with the other legs in a free and unloaded position [32], flexed, and extended their knees from 10° to 90°. During the hip test, participants lay in a supine position with their hip and knee joints flexed [33], flexed and extended their hips from 10° to 100°. During the ankle test, participants lay in a supine position with hips and knees in full extension, dorsiflexed, and plantarflexed their ankles from 5° to 30°. All the tests were measured at an angular velocity of 60°/s [34]. Three trials were conducted. The participant had at least a 2-min break between 2 consecutive trials. The strength was normalized by the body mass of each participant. A lower value indicates less strength.

### Berg Balance Scale (BBS) Assessment

A stopwatch, a ruler, a chair, and a one-step staircase were used to detect the BBS score [35], which has shown excellent reliability (ICC = 0.98) [35]. The BBS test consists of 14 items, each item was scored from 0 to 4, and the 14 items added up to a total score of 0 to 56. A higher score indicates better balance control.

### Data Reduction

The Proprioception threshold and joint ROM are calculated as the mean of three successful trials; the plantar tactile sensation was measured as the minimum monofilament gauge each participant detected correctly; the BBS score is the total of the 14 items; and the highest peak torque among the three successful trials was used and normalized by the body weight with the following equation:

$$\begin{gathered} Normalized{\text{ }}strength\left( {N \cdot /kg} \right) = \hfill \\\,\,\,\,\,\,\,\,\,\,\,{\text{ }}peak{\text{ }}torque\left( {N \cdot {\text{m}}} \right)/body{\text{ }}weight\left( {kg} \right). \hfill \\ \end{gathered}$$

### Statistical Analysis

The normality of the data was analyzed using the Shapiro-Wilk tests. Descriptive analyses were presented as mean ± standard error in all measured variables. Independent sample t-test (normally distributed) or Mann-Whitney U test (non-normally distributed) was used to compare the baselines between the two groups. Then, a Pearson (normally distributed) or Spearman (non-normally distributed) correlation was used to determine the relationship of the BBS score with each of the proprioception, PTS, pain score, ROM, and strength variables while controlling for age and BMI. After that, exploratory factor analysis was carried out among each category of the variables of interest. Finally, multivariable linear regression was used to explore the degrees of correlation between each generated factor and BBS score while controlling for age and BMI. The thresholds for the correlations coefficient (r) were as follows: trivial: 0–0.1; weak: > 0.1–0.3; moderate: > 0.3–0.5; strong: > 0.5 [36]. All analyses were conducted in SPSS 27.0 (IBM Corp.; Chicago, IL, USA) or SAS 9.4 (SAS; Cary, NC, USA). The significant level was set at α = 0.05.

## Results

Shapiro–Wilk tests showed that pain score, ROM of hip flexion, hip extension, and ankle dorsiflexion, and strength of hip flexion, knee flexion, ankle plantarflexion, and dorsiflexion were normally distributed, while others were non-normally distributed. The age, height, body mass, and BMI between the two groups were compared using Mann-Whitney U tests. Significant baseline differences were detected in weight and BMI (Table 1).

**Table 1:** Basic information of the participants (mean ± S.D.).

	KOA group (*n* = 124)	Control group (*n* = 116)	*p*
Age (years)	68.8 ± 4.0	67.9 ± 3.5	0.077
Gender	84 F, 40 M	64 F, 52 M	--
Height (am)	160.8 ± 7.5	162.4 ± 6.8	0.081
Body mass (kg)	69.3 ± 10.1	64.5 ± 9.8	< 0.001
BMI (kg/m^2^)	26.8 ± 3.2	24.4 ± 3.3	< 0.001
Affected leg	59 L, 65R	--	--
K/L radiographic grade	29 I, 35 II, 36 III, 24 IV	--	--

Values are presented as mean ± standard deviation

KOA, F, M, R, L, BMI, K/L, I, II, III, and IV represent the abbreviation of knee osteoarthritis, female, male, left, right, body mass index, Kellgren/Lawrence, I grade, II grade, III grade, and IV grade

The variables of age, height, weight, and BMI between the two groups were compared using Mann-Whitney U tests

The mean, standard deviation, and P value between the two groups in proprioception, PTS, pain score, ROM, and strength are shown in Table 2. Hip flexion/extension ROM, ankle dorsiflexion ROM, hip flexion strength, knee flexion strength, and ankle plantarflexion/dorsiflexion strength were analyzed by Independent sample t-test, while others by Mann-Whitney U tests. Significant differences were detected in BBS, all of the proprioception, and most of the PTS, ROM, and strength variables.

**Table 2:** Comparison of KOA group and control group in BBS, proprioception, PTS, pain, ROM, and strength (mean ± S.D.).

		KOA group		Control group		*P*-value	Cohen’s d
		Mean	SD	Mean	SD		
BBS		52.63	3.05	53.85	1.78	**0.008**	**0.489**
Proprioception (°)	Knee flexion	3.51	2.92	2.76	1.77	**0.016**	**0.311**
	Knee extension	4.53	3.66	3.13	2.23	**0.001**	**0.462**
	Ankle plantarflexion	4.38	4.13	3.24	3.01	**0.012**	**0.315**
	Ankle dorsiflexion	4.99	4.53	2.97	2.43	**< 0.001**	**0.556**
PTS (gauge)	Great toe	4.51	0.63	4.25	0.56	**0.002**	**0.436**
	1st metatarsal	4.50	0.63	4.29	0.63	**0.005**	**0.333**
	5th metatarsal	4.68	0.70	4.36	0.47	**< 0.001**	**0.537**
	Arch	4.60	0.69	4.44	0.52	0.053	0.262
	Heel	4.91	0.76	4.60	0.57	**< 0.001**	**0.461**
Pain		5.20	1.76	--	--	--	--
ROM (°)	Hip flexion	108.5	10.8	111.3	7.6	0.107*	0.300
	Hip extension	18.7	5.2	20.1	3.2	0.014*	**0.324**
	Knee flexion	111.6	10.4	126.5	8.5	**< 0.001**	**1.129**
	Ankle plantarflexion	41.1	7.2	49.8	5.4	**< 0.001**	**1.367**
	Ankle dorsiflexion	25.4	6.8	27.5	4.8	0.005*	**0.357**
Strength (N·m/kg)	Hip flexion	0.63	0.24	0.80	0.13	< 0.001*	**0.881**
	Hip extension	0.68	0.37	0.86	0.16	**< 0.001**	**0.631**
	Knee flexion	0.51	0.18	0.66	0.14	< 0.001*	**0.930**
	Knee extension	0.78	0.31	0.95	0.20	**< 0.001**	**0.652**
	Ankle plantarflexion	0.46	0.20	0.38	0.15	0.002*	**0.453**
	Ankle dorsiflexion	0.22	0.07	0.22	0.07	0.590*	0.000

KOA, BBS, PTS, and ROM represent the abbreviation of knee osteoarthritis, Berg Balance Scale, plantar tactile sensation, and range of motion

Bold: Significant difference between KOA and control groups

*: analyzed by Independent sample t-test, others by Mann-Whitney U tests

The correlations of the BBS with each of the proprioception, PTS, pain, ROM, and strength variables are shown in Table 3. In the KOA group, the BBS was weakly to moderately correlated with all of the variables in proprioception, PTS, pain, ROM, and strength, except for ROM of ankle dorsiflexion. While in the control group, the BBS was correlated with proprioception, and strength of knee extension, ankle dorsiflexion, and plantar flexion.

**Table 3:** Partial correlations of BBS with proprioception, PTS, pain, ROM, and strength

Variables		BBS (KOA group, *n* = 124)		BBS (Control group, *n* = 116)		
		*r*	*p*	*r*	*p*	
Proprioception (°)	Knee flexion	-0.399	< 0.001	-0.281	0.002	
	Knee extension	-0.332	< 0.001	-0.207	0.026	
	Ankle plantarflexion	-0.501	< 0.001	-0.379	< 0.001	
	Ankle dorsiflexion	-0.409	< 0.001	-0.336	< 0.001	
PTS (gauge)	Great toe	-0.284	0.001	-0.090	0.338	
	1st metatarsal	-0.283	0.001	-0.124	0.186	
	5th metatarsal	-0.197	0.028	-0.004	0.967	
	Arch	-0.270	0.002	-0.063	0.504	
	Heel	-0.291	0.001	-0.020	0.833	
Pain		-0.340 *	< 0.001	--	--	
ROM (°)	Hip flexion	0.508	< 0.001	0.158 *	0.094	
	Hip extension	0.227 *	0.012	0.165 *	0.080	
	Knee flexion	0.212 *	0.019	0.111	0.234	
	Ankle plantarflexion	0.276	0.002	0.104	0.266	
	Ankle dorsiflexion	0.145 *	0.112	0.115 *	0.219	
Strength (N·m/kg)	Knee flexion	0.336	< 0.001	0.180 *	0.055	
	Knee extension	0.309	< 0.001	0.212	0.022	
	Hip flexion	0.236	0.008	0.140 *	0.137	
	Hip extension	0.263	0.003	0.133	0.156	
	Ankle plantarflexion	0.316	< 0.001	0.410 *	< 0.001	
	Ankle dorsiflexion	0.281	0.002	0.296 *	0.001	

KOA, BBS, PTS, and ROM represent the abbreviation of knee osteoarthritis, Berg Balance Scale, plantar tactile sensation, and range of motion;

r: correlations coefficient;

*: analyzed by Pearson correlations, others by Spearman correlations;

Correlations were adjusted for age and BMI;

Lighter or darker shaded cells represent weak or moderate correlations

The factor loading for all the variables of proprioception, PTS, ROM, and strength in the two groups is shown in Table 4. Factor 1, Factor 2, Factor 3, and Factor 4 were the summaries of strength, PTS, proprioception, and ROM, respectively, with a Kaiser Meyer Olkin value of 0.809 and 0.702 in the KOA and control groups, and sphericity of < 0.001 for both groups. Pain is also used as a factor (Factor 5) in the KOA group.

**Table 4:** Factor loading for the variables among the categories of proprioception, PTS, ROM, and strength in the KOA and control groups

		KOA group (*n* = 124)					Control group (*n* = 116)			
		Factor 1	Factor 2	Factor 3	Factor 4		Factor 1	Factor 2	Factor 3	Factor 4
		(Strength)	(PTS)	(Proprioception)	(ROM)	(Strength)		(PTS)	(Proprioception)	(ROM)
Strength (*N*·m/kg)	Hip flexion	0.779	--	--	--		--	--	0.764	--
	Hip extension	0.696	--	--	--		--	--	0.622	--
	Knee flexion	0.854	--	--	--		--	--	0.864	--
	Knee extension	0.798	--	--	--		--	--	0.869	--
	Ankle plantarflexion	0.878	--	--	--		--	--	0.593	--
	Ankle dorsiflexion	0.831	--	--	--		--	--	0.508	--
PTS (gauge)	Great toe	--	0.808	--	--		--	0.565	--	--
	1st metatarsal	--	0.843	--	--		--	0.523	--	--
	5th metatarsal	--	0.855	--	--		--	0.660	--	--
	Arch	--	0.809	--	--		--	0.553	--	--
	Heel	--	0.778	--	--		--	0.747	--	--
Proprioception (º)	Knee flexion	--	--	0.875	--		0.773	--	--	--
	Knee extension	--	--	0.846	--		0.808	--	--	--
	Ankle plantarflexion	--	--	0.881	--		0.799	--	--	--
	Ankle dorsiflexion	--	--	0.809	--		0.832	--	--	--
ROM (º)	Hip flexion	--	--	--	0.764		--	--	--	0.625
	Hip extension	--	--	--	0.697		--	--	--	0.649
	Knee flexion	--	--	--	0.613		--	--	--	0.761
	Ankle plantarflexion	--	--	--	0.631		--	--	--	0.690
	Ankle dorsiflexion	--	--	--	0.521		--	--	--	0.551

KOA, BBS, PTS, and ROM represent the abbreviation of knee osteoarthritis, Berg Balance Scale, plantar tactile sensation, and range of motion

--: Factor loading < 0.5

Figure captains

In the fitted model of KOA and control groups, the overall adjusted r^2^ was 0.612 and 0.464, indicating that 61.2% and 46.4% of the variance in balance control can be explained by these five factors, suggesting that the models were well fitted; Durbin-Watson is 1.171 and 1.639, indicating that there is no autocorrelation in each model, i.e. the models were well constructed; Sig. F Change were all less than 0.001, indicating that the models are meaningful.

1 $$\begin{gathered}BB{S_{(KOA)}} = 54.41 + \left( {0.668*strength} \right) - \left( {0.579*PTS} \right) \hfill \\\,\,\,\,\,\,\,\,\,\,\,\,\,\,\,\,\,\,\,\, - \left( {1.141*proprioception} \right) + \left( {1.054*{\text{ }}ROM} \right) \hfill \\\,\,\,\,\,\,\,\,\,\,\,\,\,\,\,\,\,\,\,\,\, - \left( {0.339*pain} \right) \hfill \\ \end{gathered}$$

The equation indicated that during the stepwise backward elimination procedure of BBS as a function of balance control in the KOA group, no factors were excluded with p-values for strength, PTS, proprioception, ROM and pain of 0.003, 0.008, < 0.001, < 0.001, and 0.009, respectively. Strength and ROM increased BBS with magnitudes of 0.218 and 0.343, while PTS, proprioception threshold, and pain decreased BBS with magnitudes of 0.189, 0.374, and 0.195.

2 $$\begin{gathered}BB{S_{(control)}} = {\text{ }}53.85 + \left( {0.441*strength} \right) \hfill \\\,\,\,\,\,\,\,\,\,\,\,\,\,\,\,\,\,\,\,\,\,\,\, - \left( {0.677*proprioception} \right) \hfill \\ \end{gathered}$$

During the stepwise backward elimination procedure of BBS as a function of balance control in the control group, factors of PTS and ROM were excluded with p values of 0.706 and 0.103. Strength and proprioception have remained with p-values of 0.004 and < 0.001. The strength increased BBS with an amplitude of 0.248 and proprioception threshold decreased BBS with an amplitude of 0.374.

Equation 1 indicated that the BBS was correlated with five factors in the order of proprioception, ROM, strength, pain, and PTS in the KOA group. Equation 2 indicated that the BBS was correlated with two factors in the order of proprioception and strength in the control group.

## Discussion

This study compared group differences of older adults with and without KOA and investigated the magnitude and sequence of correlation of balance control with proprioception, PTS, pain, ROM, and strength. The outcomes partially supported hypotheses # 1 and # 2 and rejected hypothesis # 3.

This study compared proprioception, PTS, ROM, and strength between older adults with and without KOA. Most of our findings were consistent with previous studies [8–11]. Worse proprioception was detected among KOA patients. As the deterioration of articular cartilage, the proprioceptors and nerves in the articular cartilage of KOA patients are damaged, thus affecting the transmission of proprioceptive signals to the central nervous system (CNS) [8]; Worse PTS was detected, which may be due to the KOA-induced reduction in the number and function of tactile vesicles [37]; Smaller ROM was detected, which may be due to the contraction of the joint capsule and the osteophytes at the joint edges [12]; Less strength was detected, which may be due to the prolonged joint immobilisation and muscle atrophy [18].

This study indicated that proprioception was correlated with balance control, and ranked 1st among older adults with and without KOA, which was consistent with previous studies on individuals with [38] or without KOA [39]. The largest contribution of proprioception was supported by previous studies, which indicated proprioceptive afferents from the lower extremity are the primary source of information for balance control [40], and the influence of proprioception on balance control among older adults is greater than that of other sensory systems [34]. Proprioceptive afferents from muscles, tendons, and joint capsules provide information about body position and movement in space [41], and links to motor commands for precise motor control [42]. In the elderly population, age-related proprioceptor degradation is a susceptible factor for balance control [38], which may reduce the accuracy of postural signals transmitted to the CNS. This study further indicated that balance control was correlated with ankle proprioception at a higher level than with knee proprioception, which may be related to ankle strategy, which is used first to maintain body stability when external disturbances occur [43].

This study indicated that strength was correlated with balance control, and ranked 3rd and 2nd among older adults with and without KOA, respectively. Our findings were supported by previous studies, in which significant correlations of balance control to strength were detected [44, 45]. Lower limb muscles contract under the regulation of the CNS to generate corrective torques to maintain joint stability and balance control [46]. This study further indicated that (1) Knee extension strength and ankle strength were correlated with balance control in both groups. Knee extensors provide eccentric torques needed during the loading phase of the gait cycle [45] and can affect balance control [38]. Plantar flexors contract during the push-off or forward propulsion phases of gait to transfer self-generated energy to the trunk to provide support and forward propulsion, and to help initiate the swing phase [44], while dorsiflexors contract to move the center of mass anteriorly and provide stability during locomotion [45]. (2) Compared with dorsiflexion strength, plantarflexion strength has a higher level of correlation with balance control in both groups. Ankle plantar flexors, as well as knee extensors, resist the knee adduction moment in the frontal plane during the stance phase of gait and thus prevent increased weight bearing on the medial knee joint, contributing to knee stability [44]. (3) The correlation of balance control with hip strength was detected in the KOA group, but not in the control group [47]. A reasonable explanation may be that when knee and ankle strength is insufficient, the hip strength is used to compensate to maintain balance control [47].

This study indicated that ROM was correlated with balance control, and ranked 2nd among patients with KOA. The correlation of ROM with balance control is supported by previous studies. Holla et al. indicated that the reduction of ROM may lead to decreased balance control [48]. Bade et al. detected a moderate correlation between knee and hip ROM with functional impairment, and they believed that limited hip and knee ROM was one of the main causes of motor and balance dysfunction [49]. Adequate ROM allows for effective joint movement and postural adjustment [50]. Our study further indicated that (1) Among individuals with KOA, balance control was correlated with ROM of plantarflexion, but not that of dorsiflexion. Seong-Gil et al. supported our findings by showing significant correlations of postural sway to the ROM of plantarflexion, but not to dorsiflexion [47]. Plantarflexion, rather than dorsiflexion, is required during the push-off or forward propulsion phase of gait to provide the joint space needed for the push-off movement [45]. (2) No correlation was detected between ROM and balance control in the control group. Joint ROM was greater in the control group compared to the KOA group, which may be sufficient for joint rotations.

This study indicated that pain was correlated with balance control, and ranked 4th among patients with KOA. Pain is one of the main clinical symptoms of KOA and affects individuals’ functional performance [51]. Takacs et al. detected a moderate correlation between pain and balance control among KOA patients, which is consistent with this study [11]. Pain-induced joint immobilization results in muscle atrophy and limited ROM, which reduces knee stability and impaired balance control [12]. Furthermore, studies have reported that KOA patients alter their gait strategies to avoid pain, which may affect physical stability [51].

This study indicated that PTS was correlated with balance control, and ranked last among patients with KOA. To our knowledge, no previous studies investigated the correlation between PTS and balance control among patients with KOA. One previous study indicated that PTS has no relationship with BBS among older adults without KOA [39]. The difference in correlations between PTS and balance control among older adults with and without KOA may be explained by sensory reweighting. Balance relies on several types of sensory information, including somatosensory senses such as tactile sensation and proprioception [52]. In general, proprioception is transmitted via type Ia and type II sensory neurons, whereas PTS is transmitted via type III sensory neurons [53]. Alternative sensory inputs can be used to compensate for the impairments of particular sensory inputs [39]. We assume that PTS compensated for the deteriorated proprioception and provided useful information on balance control among older adults with KOA. Although a significant correlation was detected, PTS ranked last among the five factors. Previous studies indicated that PTS is important to static balance control, rather than dynamic balance control [39], since the loss of PTS sensitivity may be compensated by other sensorial systems [22]. Of the 14 BBS tests, five tested static balance control (standing unsupported, standing with eyes closed, standing with feet together, standing on one foot, and placing one foot in front of the other) [54], while the other nine tested dynamic balance control, which may explain the relatively small contribution of the PTS to the BBS. In addition, all BBS tests were conducted with shoes on, which may block the tactile signals from the ground [55].

This study provides a scientific basis for the development of KOA rehabilitation programs. We confirmed that proprioception plays the most important role in balance control, suggesting proprioceptive training should be prioritized when designing KOA rehabilitation programs. In addition, joint ROM training should be a second consideration as it plays a key role in balance control. Muscle strengthen and pain relief are also beneficial in improving balance control among patients with KOA. Tactile sensation training could be considered as a complement to proprioceptive training in KOA rehabilitation programs until proprioception is well recovered. KOA rehabilitation programs could be designed in the sequence of improving proprioception, joint ROM, strength, pain, and tactile sensation. Future studies are encouraged to compare the effectiveness of programs designed in this manner and traditional KOA rehabilitation programs.

There are limitations in this study. First, this study examined the relationship of balance control to proprioception, PTS, pain, ROM, and strength only. Other contributors, such as visual and vestibular sensations, could also influence balance control. Second, only patients with unilateral KOA were included in this study; therefore, the findings cannot be generalized to patients with bilateral KOA. Third, as a cross-sectional study, this study may involve participant selection bias. Forth, the participants were all from the community, so the findings of the study should be applied with caution to other populations.

## Conclusion

Worse proprioception and PTS, smaller ROM, and less strength were detected among older adults with KOA. Among older adults with KOA, balance control was sequentially correlated with proprioception, ROM, strength, pain, and PTS. Precise KOA rehabilitation programs may be proposed following the sequence of improving the five factors.

## Data Availability

The datasets used and/or analyzed during the current study are available from the corresponding author upon reasonable request.

## Abbreviations

BBS: Berg Balance Scale
CNS: Central nervous system
ICC: Intra-class correlation coefficients
KOA: Knee osteoarthritis
PTS: Plantar tactile sensation
ROM: Range of motion
VAS: Visual Analogue Scale
